# Exclusive endoscopic tympanoplasty efficacy in the treatment of cholesteatoma without mastoid involvement

**DOI:** 10.1007/s00405-024-08778-8

**Published:** 2024-09-26

**Authors:** Luca Bianconi, Stefano Meneghesso, Valerio Arietti, Giacomo Leonardi, Daniele Monzani, Luca Sacchetto

**Affiliations:** 1 University of Verona, Via dell’Artigliere 8, Verona, 37129 Italy; 2 Otolaryngology-Head and Neck Surgery Department, University Hospital of Verona, Piazzale Ludovico Antonio Scuro 10, Verona, 37100 Italy

**Keywords:** Endoscopic ear surgery, Middle ear, Cholesteatoma, Recurrence, Second-look operation

## Abstract

**Purpose:**

The purpose of this study is to determine the recurrence rate of cholesteatoma in patients who have undergone exclusive endoscopic tympanoplasty at our tertiary referral institution. A secondary objective is to analyze different clinical aspects that could be considered risk factors for recurrence to establish if it is possible to determine when a second-look procedure is indicated instead of a clinical follow-up.

**Methods:**

A retrospective study was performed on patients who had undergone exclusive endoscopic tympanoplasty for cholesteatoma in the last eight years and who were followed up for at least one year. The efficacy of the treatment performed only with the exclusive endoscopic technique was analyzed. Then, the anamnestic and intraoperative data were studied to identify possible factors that could increase the risk of recurrence.

**Results:**

The recurrence rate (14.5%) in patients (164) who underwent primary surgery with the exclusive endoscopic technique between January 2014 and January 2022 was similar to that in patients who underwent the microscopic technique with mastoidectomy in literature. In addition, we analyzed several clinical factors such as age, ossicular chain erosion, extension and localization of the cholesteatoma finding that only the last one could potentially be a risk factor for recurrence in this selected population.

**Conclusion:**

Exclusive endoscopic tympanoplasty has been shown to be effective in removing cholesteatoma in patients without evidence of mastoid involvement, with recurrence rates comparable to traditional microscopic technique and a minimally invasive approach, even considering the patient’s age, ossicular chain erosion and extension of the disease.

## Introduction

Cholesteatoma is a benign disease of the middle ear that is classically referred to as “skin in the wrong place” [1]. In fact, it is an accumulation of keratinous material originating from a detached epithelium and localized in the middle ear and/or mastoid. The ectopic localization of this tissue is almost always accompanied by severe inflammation, which impairs the function of the middle ear and promotes bone erosion of the surrounding structures. If this lesion is not treated, it can have serious consequences that are potentially fatal, such as meningitis or brain abscesses [2–4]. 

Radical treatment of cholesteatoma is purely surgical and requires adequate access to the structures of the middle ear to achieve complete removal of the disease. One of the main problems in the postoperative treatment of patients is the significant recurrence rate of the disease, which can occur in two ways: by the remaining diseased tissue slowly starting to grow again (residual cholesteatoma), or by an altered physiology of the middle ear, often due to a dysfunction of the Eustachian tube, which favors the formation of a new cholesteatoma (recurrent cholesteatoma). The latter can be detected by visualizing newly formed retraction pockets on the membrane.

For this reason, appropriate follow-up plays a fundamental role in the early detection of recurrence. Although still controversial, follow-up can be performed through clinical and imaging surveillance as well as a second surgical procedure, the so-called second-look surgery, usually 6–12 months after the initial procedure [5]. 

The surgical treatment of cholesteatoma has traditionally evolved with the use of the microscope. However, in order to properly visualize the structures of the middle ear with this instrument, a transmastoid approach is often necessary, requiring a retroauricular incision. Thanks to technological advances, more and more otosurgeons are using the endoscopic approach for the treatment of cholesteatoma, which allows excellent transcanal visualization of the middle ear without the need for external incisions. With increasing experience in the use of this technique, the number of patients treated exclusively with an endoscopic transcanal approach is therefore also increasing [6].

The advantages of endoscopic access in the treatment of cholesteatoma have been reported in the literature for many years and it has been shown that preservation of the mastoid allows maintenance of adequate middle ear physiology [6, 7]. 

Nevertheless, there is a need to further investigate the indications for exclusive endoscopic transcanalar treatment of cholesteatoma and to treat a larger number of patients with this technique to fully compare it with the microscopic approach.

This study has two main objectives. The first is to determine the recurrence rate of cholesteatoma in patients treated exclusively with endoscopic surgery at the University Hospital of Verona. In parallel, we will describe the surgical steps of this procedure and analyze how the use of this technique, both in a primary and second-look procedure, may affect the follow-up of the patients’ clinical history.

The second objective is to analyze the preoperative clinical characteristics, the intraoperative findings and the surgical techniques used in these patients in order to identify possible risk factors for recurrence and thus define the best possible conditions for a clinical follow-up or a second-look procedure.

## Materials and methods

The study was conducted by retrospectively analyzing data from patients who underwent surgery at the University Hospital of Verona between January 2014 and January 2022. Patients diagnosed with cholesteatoma who underwent a first tympanoplasty with an exclusively endoscopic technique and who were followed up for at least one year were included in the study. The rates of recurrent and residual cholesteatoma were therefore calculated on this population. The exclusion criteria were:

- Tympanoplasty performed for a condition other than cholesteatoma (histopathological analysis)
- Microscopic surgery or combined microscopic-endoscopic surgery
- Patients with a history of one or more previous surgical procedures in other hospitals
- Patients with a follow-up of less than one year

Based on this selected population, it was decided to study a specific subgroup of patients, namely those who underwent second-look surgery after the first exclusive endoscopic procedure.

The endoscopic technique used in the second-look surgery was as follows:

- Elevation of the tympanomeatal flap (maintaining tympanic membrane reconstruction when possible),
- Detachment of the tympanic membrane from the ossicular chain (if possible, maintaining the ossiculoplasty),
- Achievement of complete visualization of the middle ear,
- Removal of pathological tissue, if present,
- Endoscopic inspection, including an angled endoscope to visualize all hidden areas,
- Repositioning of the tympanomeatal flap.

Firstly, recurrence rates were calculated, distinguishing between recurrent and residual cholesteatomas. The patients’ medical records were then analyzed to collect specific information: age, gender, comorbidity, previous otological disease, location and extent of cholesteatoma, entity of ossicular erosion, whether ossiculoplasty was performed, and imaging findings. Based on these characteristics, the rates of recurrence and residual cholesteatoma were then calculated.

This process was important to verify whether a particular feature correlated with a higher or lower recurrence rate for each type of cholesteatoma.

## Results

For this study, 164 patients were selected, so that we treated a total of 172 ears.

Of these patients, 56 (34.1%) underwent a second-look procedure, totaling 58 ears.

Ninety-nine (99, 57.5%) of the treated ears were from male patients and seventy-three (73, 42.5%) from female patients. The age ranged from 3 to 86 years, and the average time between the first and second procedure was 18.5 months (537.0 days) with a range of 8.8 to 49.5 months.

At the second-look procedure, 25 cholesteatoma recurrences were detected (14.5% of all ears examined and 43.1% of patients who underwent the second procedure). Of these, 22 were residual (12.8% of all ears) and 3 were recurrences (1.7% of all ears). Residual cholesteatoma is defined as the presence of cholesteatoma in the middle ear or mastoid without any evidence of the formation of a new retraction pocket. On the other hand, in recurrences of the disease, a clear retraction pocket of the tympanic membrane can be identified.

Of the residual cholesteatomas, 16 were in males and 6 in females. Of the recurrent cholesteatomas, 1 was found in a male and 2 in a female.

In view of the very low rate of recurrent cholesteatoma in our study, recurrence is referred to as the sum of residual and recurrent in the following data.

**Fig. 1:**
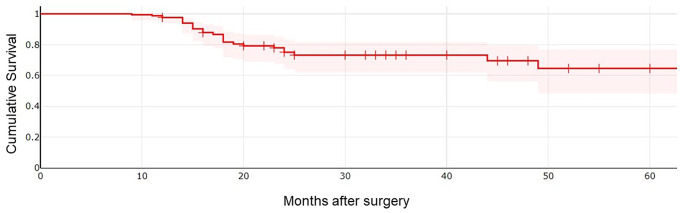
Evaluation of cholesteatoma cumulative free survival using Kaplan–Meier which indicates a 35% recidivism rate at 5 years following surgery

We estimated an incidence rate of recidivism by using a Kaplan–Meier curve. Compensating the cumulative risk with the follow-up time of the whole population, we calculated a 5-year recidivism rate of 35% (Fig. 1).

An overview of the most important clinical and intraoperative factors is presented below.

### Age

A total of 49 (28.5%) ears were from pediatric patients (3–17 years old) and 123 (71.5%) from adult patients. The recurrence rate was slightly higher in the pediatric population than in the adult population (18.3% vs. 13%), but the difference was not statistically significant (χ2, *P* = 0.37).

### Ossicular chain erosion

In 43 (25%) of the initial tympanoplasties, no involvement of the ossicular chains was found; of these, 4 (9.3%) developed a recurrence of the cholesteatoma.

Erosion of the ossicular chain was found in 129 (75%) during the first procedure; of these, cholesteatoma was found in 21 (16.2%) cases at the second-look procedure. Of these 129 patients with initial erosion, 99 (57.6%) had intact stapes, while 30 (17.4%) had erosion of the stapes. Of the 99 patients with initial ossicular erosion in whom the stapes was spared, 16 (16.1%) were found to have recurrence at the second examination. Of the 30 patients with initial ossicular erosion that also involved the stapes, 5 (16.6%) were found to have recurrence at the second examination (Table 1).

**Table 1:** Rates of recurrence of cholesteatoma in patients with different involvement of the ossicular chain

	Number of patients	Recurrence of cholesteatoma
Without involvement of the ossicular chain	43	4 (9.3%)
With involvement of the ossicular chain	129	21 (16.2%)

These data were analyzed to determine whether ossicular chain erosion and its extent might confer a higher risk of recurrence with residual cholesteatoma, but the statistical analysis was not significant (χ2, *P* = 0.26).

### Ossiculoplasty

Of the 172 ears that were treated exclusively with endoscopic tympanoplasty, ossiculoplasty was also performed in 95 (55.3%) and not in 77 (44.7%). Of the former, 13 (13.7% of patients who underwent ossiculoplasty) developed a recurrence, while of the latter, 12 (15.6% of patients who did not undergo ossiculoplasty) developed a recurrence. This difference was not statistically significant (χ2, *P* = 0.75). Of the 95 cases in which ossiculoplasty was performed, PORP was performed in 75 cases, with recurrence occurring in 9 (12%) cases, while TORP was performed in 20 cases, with recurrence occurring in 4 (20%) cases (Table 2).

**Table 2:** Rates of recurrence of cholesteatoma in patients who underwent ossiculoplasty

	Number of patients		Recurrence of cholesteatoma	
No ossiculoplasty performed	77		12 (15.6%)	
Ossiculoplasty performed	95		13 (13.7%)	
	PORP 75	TORP 20	9 (12%)	4 (20%)

In addition, it was decided to analyze the intraoperative management of ossiculoplasty by combining these data with the degree of involvement of the ossicular chain (see Table 3). Five subgroups were thus formed:

**Table 3:** Rates of recurrence of cholesteatoma in patients with different intraoperative ossiculoplasty management

	Number of patients	Recurrence of cholesteatoma
No ossicular chain involvement	33 (19.2%)	2 (6.1%)
No ossicular chain involvement, but removal due to intraoperative necessities	2 (1.1%)	0
Involvement of the ossicular chain and no reconstruction	28 (16.3%)	7 (25.0%)
Involvement of the ossicular chain with reconstruction (ossiculoplasty)	93 (54.1%)	13 (13.9%)
Ossicular chain involvement, removal during the first surgery, ossiculoplasty during the second surgery	16 (9.3%)	3 (18.7%)

- no involvement of the ossicular chain and preservation of the chain
- no involvement of the ossicular chain, but removal due to intraoperative necessity
- involvement of the ossicular chain and no reconstruction
- involvement of the ossicular chain with reconstruction (ossiculoplasty)
- involvement of the ossicular chain, removal during the first operation, ossiculoplasty during the second operation

Although the risk of recurrence appeared to be higher in patients in whom the ossicular chain was preserved despite its involvement in the disease (25.0% versus 13.9%), the difference was not statistically significant (χ2, *P* = 0.20).

### Extension

To analyze to what degree the extension of the cholesteatoma could be a risk factor for the recurrence of the disease, it was decided to divide the tympanic cavity into six anatomical parts accessible by endoscopic technique, as well-described by Marchioni et al. [8] (i.e. epitympanum, mesotympanum, protympanum, retrotympanum, hypotympanum and antrum). The possibility of treating attic cholesteatoma with an endoscopic technique thanks to the use of angled endoscopes is extensively studied in the literature [9, 10]. If the extent and localization of the cholesteatoma were such that a complete excision could not be ensured through the endoscopic technique, the procedure was converted to a microscopic approach, and the patient was excluded from the study.

The 172 ears were therefore divided into subgroups according to the localizations affected, for each of which the recurrence rate was calculated. Forty-five of these patients (45, 26.2%) had only one affected region at initial surgery; of these, 4 (8.9%) developed recurrence. Sixty-two (62, 36.0%) had two affected regions at initial surgery; of these, 10 (16.1%) developed recurrence. Forty-five (26.2%) had three affected regions at initial surgery; of these, 6 (13.3%) developed recurrence. Thirteen (7.5%) had four affected regions at initial surgery; of these, 4 (30.7%) developed recurrence. Seven (4.1%) had five affected regions at initial surgery; of these, 1 (14.3%) developed a recurrence. No patient had all six regions affected (Table 4).

**Table 4:** Rates of recurrence of cholesteatoma in patients depending on the number of the subsites of the middle ear involved

Number of subsites involved	Number of patients	Recurrence of cholesteatoma
1	45 (26.2%)	4 (8.9%)
2	62 (36.0%)	10 (16.1%)
3	45 (26.2%)	6 (13.3%)
4	13 (7.5%)	4 (30.7%)
5	7 (4.1%)	1 (14.3%)
6	0	0

These results show that there is no statistically significant difference between the groups. Thus, there is no correlation between the extent of disease and the likelihood of recurrence (χ2, *P* = 0.20).

### Preferred localization

Of the 172 ears treated, the epitympanum was affected in 100 (58.1%) cases, the mesotympanum in 137 (79.6%) cases, the protympanum in 32 (18.6%) cases, the retrotympanum in 68 (39.5%) cases, the hypotympanum in 21 (12.2%) cases and the antrum in 27 (15.7%) cases during the first procedure. Only two cases involved the mastoid, and these were included in the study because an exclusively endoscopic approach was used in which an inside-out mastoidectomy was performed.

Second-look procedures revealed 15 recurrences in the epitympanum, 10 in the mesotympanum, 2 in the protympanum, 7 in the retrotympanum, 0 in the hypotympanum, 8 in the antrum and 5 in the mastoid.

In these cases, the cholesteatoma was already present in the epitympanum in 13 of 15 cases (86.6%), in the mesotympanum in 9 of 10 (90%), in the protympanum in 1 of 2 (50.0%), in the retrotympanum in 5 of 7 (60.0%) and finally in the antrum in 3 of 8 (37.0%) at the first examination. In the mastoid, no cholesteatoma was suspected at this level at the first tympanoplasty in all cases (Table 5).

**Table 5:** Rates of recurrence of cholesteatoma depending on which subsite of the middle ear is involved

Subsites involved	Number of patients considering the first time procedure	Recurrence of cholesteatoma in second look procedure	Presence of cholesteatoma at the same subsite during in the first look.
Epitympanum	100	15	13/15 (86,6%)
Mesotympanum	137	10	9/10 (90%)
Protympanum	32	2	1/2 (50,0%)
Retrotympanum	68	7	5/7 (71,0%)
Hypotympanum	21	0	0/0 (0,0%)
Antrum	27	8	3/ 8 (37,0%)
Mastoid	2	0	0

These results show that the areas most prone to recurrence are the epitympanum and the mesotympanum, a fact that also confirms that the epitympanum is a very difficult area to reach with the endoscope to eliminate the disease (χ2, *P* = 0.04).

### Combined technique during second look

7 out of 172 (4.7%) were treated with a combined technique (endoscopy and mastoidectomy) as part of the second-look procedure. Of these, recurrence of the cholesteatoma involving the mastoid was found in 3 cases. In the other 2 cases, the involvement of the mastoid was assessed and treated using an exclusive endoscopic technique with an inside-out mastoidectomy.

Considering that, in the selected population underwent only endoscopic tympanoplasty only 5 patients had mastoid involvement during the second-look procedure at the end of the study, representing 2.9% of all patients studied (172 ears). It is important to note that 51 patients underwent endoscopic tympanoplasty exclusively during both the first and second procedures.

## Discussion

Endoscopic surgery is one of the surgical branches that has undergone the greatest technological development in recent years. In diseases of the middle ear, such as cholesteatoma, this technique makes it possible to magnify areas that are hidden and difficult to access with microscopic techniques [11].

The endoscopic technique is known to be minimally invasive, as proved by a retrospective analysis of several cases which showed that surgeons do not need to remove excessive healthy bone to access the area, allowing also for a more careful preservation of the ossicular chain. Furthermore, the results showed that use of an angled endoscope can reduce the frequency of open techniques and decrease operation times [12].

When treating cholesteatoma with exclusive endoscopic techniques, the mastoid with all its cells can be preserved intact. The mastoid is a cavity that serves as a functional reserve (referred to as a “buffer” in the literature) to regulate middle ear pressure. If it is altered, severed or obliterated during a mastoidectomy, it loses its physiological function. This leads to altered ventilation and pressure in the middle ear, which can lead to the formation of new retraction pockets of the membrane and thus to recurrent cholesteatomas [13].

Considering the advantages of an exclusive endoscopic technique as previously explained, the rationale of this study is to review its results in terms of recurrence and compare them with those found in the literature on microscopic techniques.

This study shows that the recurrence rate (documented in a second-look surgery) in patients who underwent exclusive endoscopic tympanoplasty for cholesteatoma at our institution is 14.5%. This result confirms that the recurrence rates for this technique are similar to those found in the literature for microscopic techniques [5, 14].

The result obtained from the 5-year cumulative risk calculation with the Kaplan Meier curve (i.e. 35%) is comparable to studies which analyse similar populations in terms of size, composition and surgical treatment received [15]. 

This also confirms that the follow-up is mandatory for the right management of these patients, even if performed with a second-look procedure, and it has to be organized for at least 5 years.

An important aspect that emerged from this study is that the endoscopic technique seems to have the same efficacy in both groups, even though the literature suggests that pediatric cholesteatomas are more aggressive than adult cholesteatomas [5]. However, further studies are needed to confirm this finding.

Furthermore, while it is often hypothesized that ossicular chain erosion and preservation may increase the risk of recurrence, this does not appear to be the case with the endoscopic technique, as the calculated difference in risk is not statistically significant. The same conclusion is reached when analyzing the management of the ossicular chain with ossiculoplasty. In this study, performing ossiculoplasty in the first phase instead of the second phase does not increase the risk of recurrence with an exclusively endoscopic approach.

Regarding the involvement of different areas of the middle ear in patients with second look, we found that the most affected areas were the epitympanum (58.1%) and the mesotympanum (79.6%), which is consistent with the literature [16]. Of the 15 patients in whom the epitympanum was affected at the second examination, 13 (86.6%) had a cholesteatoma affecting the same area at the first examination. Our study confirms, as shown in the literature, that it is important to examine this region thoroughly, especially at the first but also at the second operation, as it can be difficult to visualize the entire area and remove the disease completely.

A final aspect that emerged from this study is that 2 of the patients who were operated on both the first and second time with an exclusively endoscopic technique developed a new recurrence during the follow-up. These patients had already developed a recurrence after the first operation, which was diagnosed and treated at the second look, but they developed a new recurrence with residual cholesteatoma. Due to the particular characteristics of this disease, this underlines the importance of establishing a follow-up program for these patients, even after a second-look procedure, regardless of the surgical technique.

Although the recurrence rates between endoscopic and microscopic techniques are similar [5, 14], endoscopic tympanoplasty alone allows for a less invasive procedure and thus greater sparing of the otological structure in selected patients without mastoid involvement.

The strength of this study was to include only patients who underwent a first surgery with an exclusive endoscopic technique in order to focus on the patients who underwent a second surgery also with an exclusive endoscopic technique (51 patients in total). To our knowledge, there is no other study in the literature that includes such a large number of patients who underwent both the first and second surgery with an exclusive endoscopic tympanoplasty.

In any case, it would be useful to plan a prospective and prolonged follow-up study for these patients to monitor the possible development of new recurrences in relation to the different characteristics of our study and to standardize the choice of the second procedure.

In fact, since there is a growing use of the diffusion-weighted (NON-EPI DWI) magnetic resonance imaging for surveillance after the surgical intervention, the proper management of the follow-up of these patients is still debated [17]. Even though the sensitivity of the MR is significantly high, this technique is burdened with a relevant percentage of false-negative rate, considering that the smallest cholesteatoma correctly identified by non-EPI is commonly reported to be 3 mm, and thus is burdened with a real risk of losing patient during the follow up [18]. A second look procedure, especially with the endoscopic technique, allows a prompt detection of the recidivism in the first year of follow-up, with a minimally invasive approach, when the MR is less effective [19].

Further studies are needed to identify the risk factors that expose patients to a higher risk of recurrence and to determine which patients should be candidates for clinical follow-up and which for second-look procedures.

## Conclusion

Exclusive endoscopic tympanoplasty is confirmed as a technique that can ensure recurrence rates similar to microscopic techniques in patients in whom the mastoid is unaffected. Since this technique does not affect the integrity of the mastoid, this functional structure can be preserved, resulting in a very low recurrence rate of cholesteatoma. This once again confirms the importance of the physiological function of the mastoid [13].

This study also shows that endoscopic techniques succeed in managing equally different kinds of cholesteatoma presentation even in pediatric patients, even if the ossicular chain is involved and despite the extension of the disease in the middle ear. A possible risk factor for cholesteatoma recurrence seems to be the epitympanum and mesotympanum involvement, as already known in literature. Further investigations are needed in future prospective studies to personalize treatment as much as possible, in order to establish in which patients it is possible to set up a clinical follow-up and in which ones a second look procedure is mandatory for the high risk of recurrence.

## Data Availability

Availability of data and material is possible upon reasonable request, deidentified for maintenance of anonymity.
